# Dental care for patients with down syndrome: A survey for dentists of the college of the balearic islands

**DOI:** 10.4317/jced.61747

**Published:** 2024-08-01

**Authors:** Sebastiana Arroyo-Bote, Catalina Bennasar-Verger, Ángel-Arturo López-González

**Affiliations:** 1 PhD, MD. Coordinating teacher of Conservative Dentistry. ADEMA School of Dentistry. Researcher of ADEMA Health IUNICS group. University of the Balearic Islands. Palma, Balearic Islands, Spain. Associate professor of Barcelona University. IDIBELL Researcher; 2 PhD, MD. ADEMA School of Dentistry. Researcher of ADEMA Health IUNICS group. University of the Balearic Islands. Palma, Balearic Islands, Spain

## Abstract

**Background:**

Down Syndrome (DS) presents with systemic, craniofacial and oral alterations accompanied by different levels of intellectual disability and because of this, they frequently require professional dental care. Objective: This work aims to know the dental care patients with DS receive from dentists in the Balearic Islands.

**Material and Methods:**

An 11-question survey was carried out via email from the College of Dentists of the Balearic Islands. The researchers conducted the survey based on previous researchs. The first three questions refered the professional´s profile (age, sex and years since graduation) and the restant 8 were focused on the academic training and dental care provided to patients with DS.

**Results:**

129 surveys were collected. 40.45% were between 34-43 years old, 67.84% were women, and 32.16% were men. 33.30% had been in professional practice for between 15-24 years, followed by those with 4-14 years with 27.33% and those with 25-34 years with 24.04%. 81.64% received undergraduate academic training, and 60.72% completed training after graduating. 57.17% believe that patients with DS should be treated by a dentist specialised in special patients, 20.67% by a pediatric dentist, and 18.87% by a general dentist. 63.40% perform sealing, fillings or dental extractions, 60.6% perform oral examination, oral cleaning and give prophylaxis instructions, and 26.72% state that they perform endodontic treatments. Significant differences were found between some of the variables analysed and the age, sex, academic training or professional scenario of the professionals.

**Conclusions:**

Post-graduate training increases the likelihood that dentists will feel comfortable with sealing-filling-extraction treatments by 7.48 times and endodontic treatments by 3.26 times.

** Key words:**Down Syndrome, Trisomy 21, Surveys and Questionnaires, Dental Care for Children, Oral Health.

## Introduction

Down Syndrome (DS) is the most prevalent chromosomal anomaly, affecting one of every 675 births (1). Thanks to improved health care, life expectancy has grown to over 55 years, compared to 25 in the 1980s (2). This syndrome is due to a chromosomal alteration is in pair 21, which, in 96% of cases, instead of having 2 chromosomes, has three. Apart from these trisomic forms, there are two more alterations. One is mosaicism, where only a percentage of the cells present have this trisomic alteration, in which case the trisomic alterations are much more mitigated and the other alteration is the translocation in which the number of chromosomes is normal; however, in one of them, there is an excess of genetic material, in which case there will be phenotypic manifestations only if the translocation is not compensated (3).

People with DS, apart from presenting some degree of intellectual disability, have medical, craniofacial, and oral characteristics, as it has been already mentioned.

The most common medical problems they present are muscle hypotonia, immune system alterations, lung infections, sleep apnoea, Alzheimer’s disease, congenital heart problems, haematological problems, oesophageal problems, atlantoaxial instability, seizures and diabetes (4). These alterations mean parents are advised to visit their doctors to prevent these problems (5).

Patients with DS exhibit distinctive intraoral, extraoral, and craniofacial features. Therefore, their underdevelopment of the middle third of the face results in specific phenotypic characteristics: brachycephaly, delayed closure of fontanels, hypoplasia of the bones of the face, lack of development, especially of the upper jaw, reduction and absence of the maxillary and frontal sinuses, depression of the bridge of the nose, epicanthic folds, palpebral fissures oriented obliquely upwards, hypertelorism or hypotelorism, lower and slightly oblique implantation of the ear pinnae and short, wide necks with increased subcutaneous cellular tissue.

It must be considered that these anomalies of the middle third of the face and hypotonia of the orofacial muscles can cause functional problems in breastfeeding, swallowing, chewing and speaking (6).

Due to hypotonia, the tongue tends to protrude, and then, to achieve a more stable occlusion, the jaw also protrudes. The combination of this tongue thrust and a prognathic jaw leads to open-mouth breathing, which can trigger obstructive sleep apnoea syndrome and respiratory tract infections, which are much more frequent in these patients (6,7).

Regarding intraoral problems, patients with DS are characteristic of delayed tooth eruption. They also present dental alterations in number (oligodontia in 38-63%, with the lateral incisor being the most frequent) and agenesis, in structure (hypoplasia and hypocalcification), shape (conical incisors), and position (transpositions, the most frequent being of the canine and first premolar) (8).

In 8% of the patients, an absolute macroglossia is present and in the remaining cases, we can found a relative macroglosia because the size of the oral cavity is altered due to the lack of development of the middle facial third. They usually present a fissured or scrotal tongue (9).

They also present lip fissures, angular cheilitis and bruxism.

This patient also presents sleep apnoea, something they may present oral breathing and nasal obstruction, which causes xerostomia, plaque accumulation, and alteration of the normal saliva-cleaning mechanism (9).

Regarding to dental caries incidence, we can found a discussion point, because it could be similar or lower than in the general population, and it could be explained by microdontia and the delay in the eruption of permanent teeth. This delay in eruption and the agenesis of the permanent teeth force us to adopt more extreme preventive measures in the primary dentition.

Nowadays, altered salivary mechanisms and interdental food debris can produce a lower oral pH, leading to demineralisation and a higher incidence of cavities.

In DS, a high incidence of gingivitis at an early age and periodontal diseases have been described, with periodontal destruction similar to juvenile periodontitis. Gravity does not correspond to greater plaque accumulation. Currently, it is attributed to an alteration of the immune system response (3,10). In a recent study (11), DS was strongly associated with periodontitis but moderately with gingivitis.

Malocclusions in DS are posterior crossbites, which tend to be skeletal class III (3,12), relative mandibular prognathism due to a small maxilla, and anterior open bites. Crowding is an exception since small jaws and microdontia help to avoid it.

A short cranial base causes class III, and mandibular hypoplasia is described as a characteristic of this syndrome (13).

-AIM

As a result of all these observations, the role of dentists is crucial in the inspection, examination, treatment and monitoring of patients with DS, which is why our main aim is:

To determine the level of dental care of registered dentists in the Balearic Islands provided to patients with DS.

As secondary objectives, we propose:

To determine the correlation between the treatments administered to patients with DS and the age, gender, year of graduation, undergraduate academic background, postgraduate academic background, and professional clinical experience of the healthcare provider.

## Material and Methods

The study has the approval of the Ethics Committee of the Balearic Islands, with research project nº. IB4142/20PI, approved in the session of September 9, 2020 (no. 26/20).

A survey of 11 questions was applied using the Survey Monkey platform. The College of Dentists of the Balearic Islands emailed all members (815 members) a copy.

The researchers prepared the questionnaire based on previously conducted studies in the literature (10,12). Dentists received an email in October 2020 with a survey link. The survey was anonymous, and each member could only respond to the questionnaire once, respecting data protection law. The participants’ informed consent was obtained through the first question of the questionnaire, where, after explaining the objective of the survey, voluntary participation in the research was accepted.

The survey consists of two parts. The first three questions refer to the dentist’s profile and the second part, 8 questions, relate to the dentist’s training and care with patients with DS ( Table 1). A dichotomy response (yes-no) was used in questions 4 and 5. A three-point Likert scale was used in question 6. A five-point Likert scale was used in question 7 A four-point Likert scale was used in question 8 and 11 (14). In your opinion, a child with DS should be followed up and treated for? This The questions 9 y 10 was answered with a value between 0. (not at all accessible) and 10 (very accessible) (15).

The answers “I have no opinion” and “I do not treat patients with DS” were counted as possible responses to the questions.

A descriptive statistical and the chi-squared statistical study was carried with the *p*<0,05 value for significance, and also the multivariate multinomial logistic regression test were carried out.

## Results

**Figure 1:**
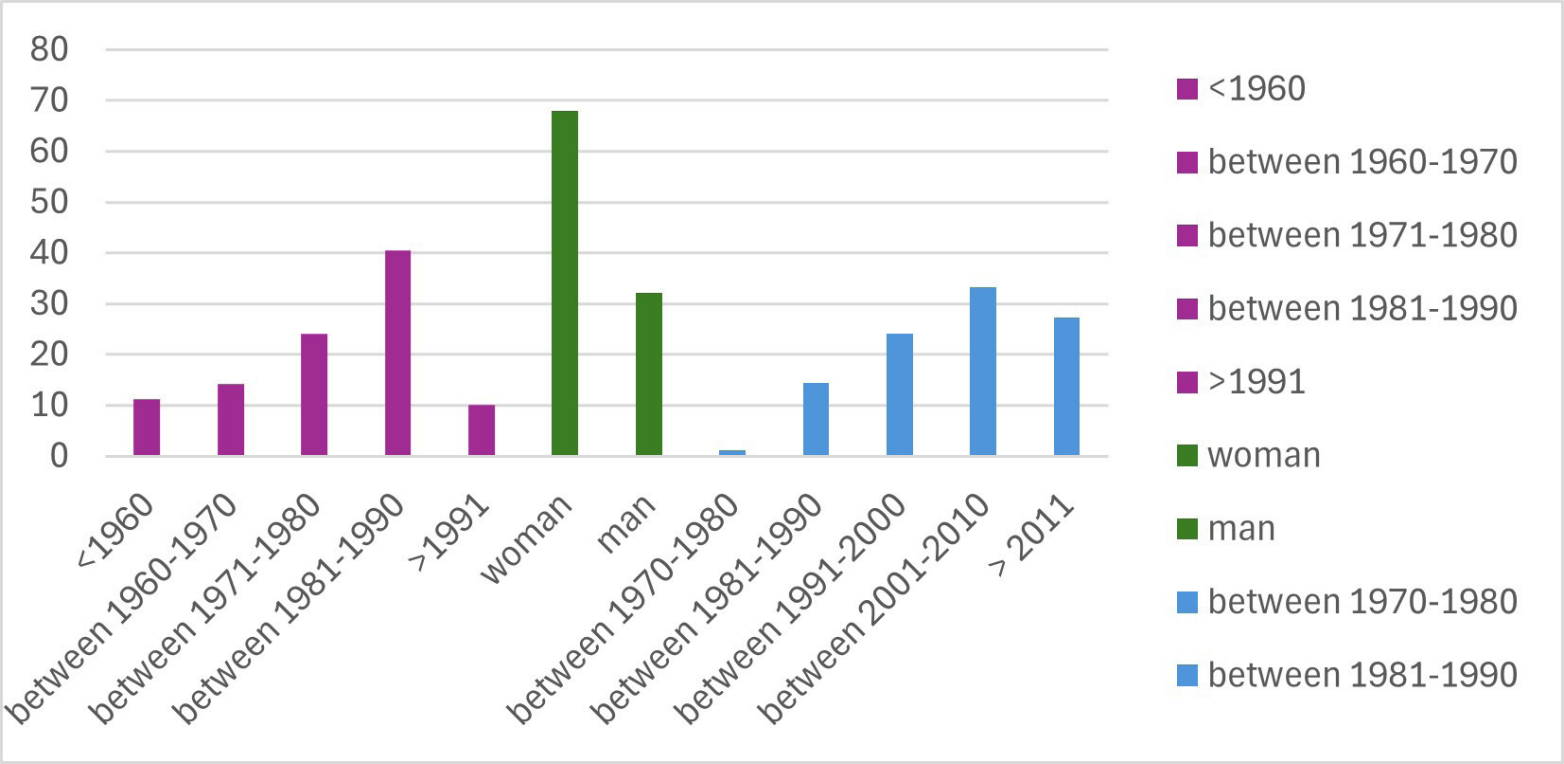
Histogram with data obtained from the responses of:1.- What year were you born? 2.-Sex 3.- Year in which you obtained the title of Medical Stomatologist or Dentist.

One hundred twenty-nine completed surveys were collected, meaning 15.94% participation. The descriptive statistical analysis of the results ( Table 1) showed that most participants (40.45%) were born between 1981-1990, 34-43 years old, followed by those were born between 1971-1980, 44-53 years old (24.13%). 67.84% were women compared to 32.16% men. 33.30% had been in professional practice for 15 to 24 years (title between 2001-2010), followed by those for less than 14 years with 27.33% (title > 2011) and those for 25-34 years with 24.04% (title between 1991-2000) (Fig. 1).

**Figure 2:**
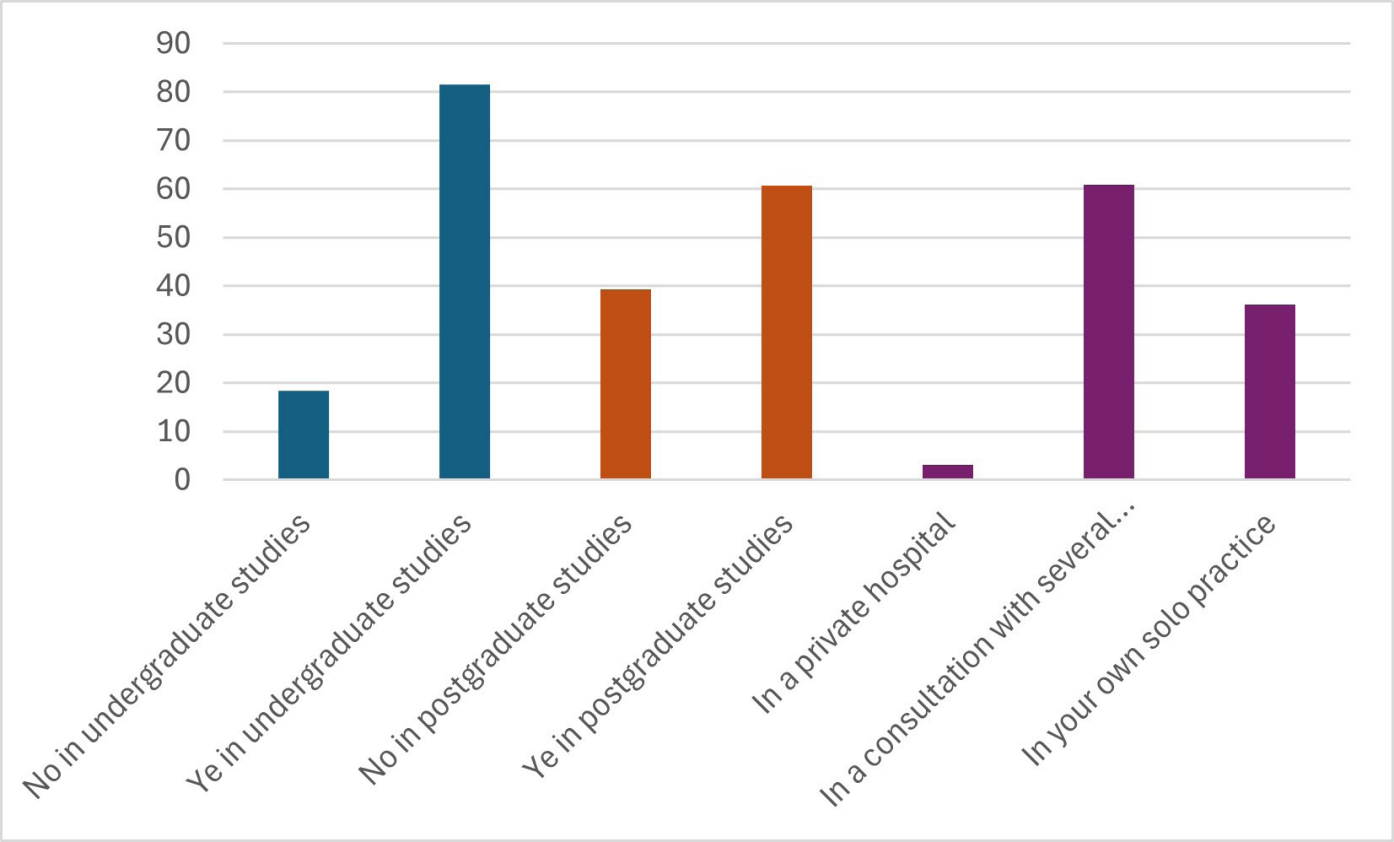
Histogram with data obtained from the responses of: 4._ In your university studies, did you receive information about patients with Down Syndrome? 5.-Have you carried out studies on treatment in special patients since your university studies? 6.- Where do you carry out your professional practice?

81.64% received academic training in special patients during undergraduate studies and 60.72% completed training after graduating. The most frequent professional scenario is a clinic with several professionals (60.84%), followed by professionals who work alone (36.10%) and those who work in hospitals (3.06%) (Fig. 2).

**Figure 3:**
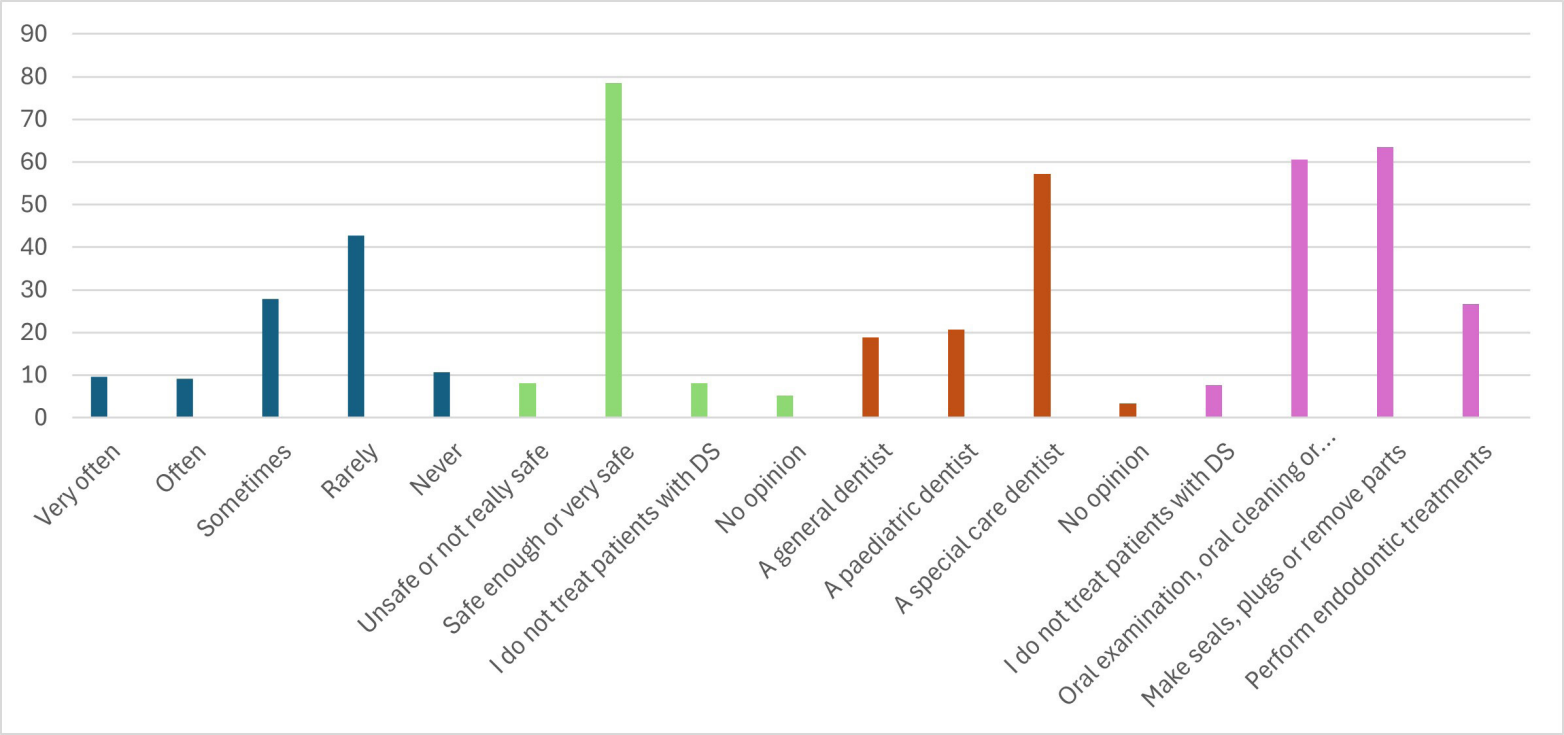
Histogram with data obtained from the responses of: 7.- How often do you treat patients with Down Syndrome? 8.- How safe do you feel treating patients with Down Syndrome? 9.- In your opinion, a child with Down syndrome should be followed and treated by.

78.47% stated that they felt safe or safe enough treating patients with DS, 8.15% indicated that they were unsure, the same percentage, 8.15%, said that they did not treat patients with DS, and 5.23%. They do not express an opinion regarding this question (Fig. 3).

Regarding the question of who should treat patients with DS, 57.17% think that a dentist specialised in special patients should do it, 20.67% think that a paediatric dentist should do it, 18.87% believe that a general dentist should do it, and 3.29% have no opinion on this issue (Fig. 3).

In answer to the multiple-response question about dental treatments, respondents stated that 63.40% performed sealing, fillings, or dental extractions in patients with DS, 60.6% performed an oral examination and oral cleaning and gave prophylaxis instructions, 26.72% stated that they performed endodontic treatments, and 7.64% said they did not treat patients with DS (Fig. 3).

A descriptive statistical analysis was carried ( Table 1). For significance, the chi-squared statistical study was carried with the *p*<0,05 value ( Table 2, Table 3) and also the multivariate multinomial logistic regression test ( Table 4) were carried out.

 Table 2 presents the statistical analyses of the results between the possible treatments, and the professionals’ age or year of graduation or the professionals’ sex. Significant differences were found in carrying out oral examination treatments, oral cleaning, or giving cleaning instructions and the year of graduation (*p*=0.04). Also significant differences were found between the sex of the participants and the sealing-filling and tooth extraction treatment (*p*=0.015).

 Table 3 presents the statistical analysis of the results between the possible treatments and undergraduate academic training or post-graduate academic training. And also, the statistical analysis in relation to the professional scenarios of the participants. the sealing-filling and tooth extraction treatment in terms of undergraduate training (*p*=0.022). Likewise, significant differences were found in post-graduate training concerning sealing-obturation-extraction treatment (*p*=0.002) and endodontic treatment (*p*=0.003). Finally Significant differences were found between professionals who work alone and those who perform endodontic treatment (*p* = 0.03).

The multivariate multinomial logistic regression study ( Table 4) shows that only postgraduate training influences the outcome, increasing the probability of feeling comfortable with sealing-filling-extraction by 7.48 times and endodontics by 3.26 times.

## Discussion

When studies regarding special patients are aimed, sometimes there are comparing patients with mental retardation mixed with patients with DS (14). To date, no research similar to this study has been conducted in our community.

In this study, a significant number of dentists (78.47%) say that when treating patients with DS, they feel enough safe or very safe, but only 18.87% think that the most appropriate dentist to treat patients with DS is the general dentist. In the study by Descamps *et al*. (14), it is clear that only 49% claim to have confidence in treating patients with DS, and only 14.5% think that the most appropriate dentist to treat patients with DS is a general dentist. In another study, where parents were asked their opinion about dental care for their child with DS (15), more than 50% responded that they had taken their child to a private dental office, and 53% of the children went to see the same dentist as their siblings. This discrepancy shows that parents are more often in favour of being treated by a general dentist, while our study reflects that 57.17% of dentists believe that special care dentists should treat patients with DS.

Weil *et al*. (12) evaluated the educational experience regarding care for patients with special needs, mental retardation, and autism. General dentists in 71% of the 500 respondents responded that they had not been sufficiently prepared to treat patients with mental retardation during their undergraduate education. These data coincide with the study by Descamps *et al*. (14). On the other hand, in our research, 81.64% stated that they had received information about the treatment of patients with DS during their undergraduate training.

Casamassimo *et al*. (10) conducted a much broader investigation into practising dentistry with special patients: cerebral palsy, intellectual disabilities, and medically compromised children. The following conclusions were drawn: in 52% of cases, dentists rarely or never treat children with intellectual disabilities, and 41% would like to receive more training. These results do not even coincide with the results of Descamps *et al*. (14), in which 78.50% rarely or never treat children with DS, and 73% of them would like to receive more training, nor with our study, where 42.75% seldom treat patients with DS, 81.64% have received information about patients with DS in their university studies, and 60.72% have carried out studies on treatment in special patients after their university studies.

Kleinart *et al*. (16) used a 10-year-old virtual patient with DS as a training tool for dental students. The students were required to make clinical decisions based on the virtual patient. Providing students with clinical experiences of unusual patients is challenging in education. However, computer software could serve as an excellent tool to enhance training. Along these lines, Mac Giolla Phadraig also defends a change in the educational program (17).

As Descamps (14) states, in addition to taking into account the two previous statements by Keinart and Mac Giolla *et al*., perhaps new strategies would be necessary for the education of our students so that these changes in the studies in this area are reflected in the treatment of patients with DS.

Sadhbh O’Rourke *et al*. (18), apart from examining, through a bibliographic review, whether education at the undergraduate and postgraduate level in the dental care of special patients increases the confidence of students and professionals, they also evaluated whether There was a correlation between greater professional confidence and higher quality of dental care for people with special needs. It was concluded that professionals with advanced theoretical and practical education showed much greater confidence in treating special patients than those who had not received it. Additionally, they were more likely to employ appropriate behaviour management techniques and treat special patients regularly.

This statement is very well reflected in our research, in which 81.64% received information during their university studies about patients with DS, 60.72% have conducted studies on treatment in special patients after their undergraduate period, and 42.75% of our dentists rarely treat patients with DS.

In the study by Descamps and Marks (19), dentists felt confident performing oral examinations, removing calculus, or giving cleaning instructions. In our study, when relating these three variables to the year of birth, graduation, or bachelor’s degree, we found significant differences in relation to performing oral examination treatments, oral cleaning, or giving cleaning instructions and the year of graduation or bachelor’s degree (*p* =0.04).

Furthermore, in the statistical analysis of the results between the possible treatments, such as sealing, fillings and extractions, and sex, undergraduate academic training and academic training after graduation, we found significant differences between the sex of the participants and the treatment of sealing-filling and tooth extraction (*p*=0.015). Likewise, significant differences were found in this same treatment concerning undergraduate training (*p*=0.022). Finally, significant differences were found in training after graduation and in relation to sealing-obturation-extraction treatment (*p*=0.002) or endodontic treatment (*p*=0.003).

In our statistical analysis in relation to the professional scenario of the participants, we found significant differences between professionals who work alone and endodontic treatment (*p*= 0.03). Yap *et al*. (20), through a study through a survey, reported that the reason for not performing endodontic treatments in these patients is limited cooperation, poor dental hygiene and uncontrolled movements. They concluded that endodontic treatment in special patients must be performed by specialists who know how to use the pharmacological approach better to control behaviour and perform treatments with a simple visit compared to the general dentist.

After our statistical analyses, we can say that the dentists in our survey are not limited to performing oral examinations, removing calculus, or giving cleaning instructions. This could be supported by the 81.64% of the dentists in the College of Dentists who claim to have received information in their university studies about patients with DS, 60.72% to have carried out studies on treatment in special patients after their university studies, and 42.75% (this percentage is higher in the other studies we have referred to) of our dentists rarely treat patients with DS.

There has certainly been an increased emphasis in recent years on providing dentistry students worldwide with education in the dental care of special patients. However, several barriers remain to providing a comprehensive education in the area (18).

These barriers have been categorised by Ettinger (21) in a review of barriers to teaching dental care to geriatric and special patients:

a. Inadequate curricular time: lack of time in the clinical curriculum for new disciplines.

b. Inadequate funding, including insufficient resources to support senior and special care clinics.

c. Lack of teachers trained as teachers for didactic and clinical courses (21,22).

These barriers may prevent dental professionals from receiving adequate education regarding treating the most vulnerable members of our society.

## Conclusions

With the limitations of this study, we can conclude:

1. 78.47% of dentists in the Balearic Islands feel enough safe or very safe when treating patients with DS.

2. 57.17% of dentists in the Balearic Islands consider that patients with DS should be treated by a dentist specialising in special patients.

3. There are significant differences in performing oral examination treatments, cleaning or giving cleaning instructions and the year of graduation or bachelor’s degree (*p*=0.04).

4. Significant differences were found between the sex of the participants, undergraduate training and training after graduation or bachelor’s degree in relation to sealing-filling and tooth extraction treatment (*p*=0.015, *p*=0.022 and *p*=0.002).

5. There are significant differences in the group of dentists who practise independently regarding endodontic treatment (*p*=0.03) as well as in the group of dentists with postgraduate training in endodontic treatment (*p*=0.003).

6. Post-graduate training increases the likelihood that dentists will feel comfortable with sealing-filling-extraction treatments by 7.48 times and endodontic treatments by 3.26 times.

## Figures and Tables

**Table 1:** The survey with the descriptive statistical analysis of the results The first three questions refer to the dentist’s profile: year of birth, sex and year of graduation, and the others eight questions, relate to the dentist’s training and care with patients with DS.

Questions	Answer	Result %
1.- What year were you born?	<1960 between 1960-1970 between 1971-1980 between 1981-1990 >1991	11.15 14.21 24.13 40.45 10.06
2.-Sex	woman man	67.84 32.16
3.- Year in which you obtained the title of Medical Stomatologist or Dentist	between 1970-1980 between 1981-1990 between 1991-2000 between 2001-2010 > 2011	1.04 14.29 24.04 33.3 27.33
4.- In your university studies, did you receive information about patients with Down Syndrome?	No Yes	18.36 81.64
5.- Have you carried out studies on treatment in special patients since your university studies?	No Yes	39.28 60.72
6.- Where do you carry out your professional practice?	In a private hospital In a consultation with several professionals In your own solo practice	3.06 60.84 36.1
7.- How often do you treat patients with Down Syndrome?	Very often Often Sometimes Rarely Never	9.65 9.11 27.81 42.75 10.68
8.- How safe do you feel treating patients with Down Syndrome?	Unsafe or not really safe Safe enough or very safe I do not treat patients with DS No opinion	8.15 78.47 8.15 5.23
9.- In your opinion, a child with Down syndrome should be followed and treated by:	A general dentist A paediatric dentist A special care dentist No opinion	18.87 20.67 57.17 3.29
10.- In your opinion, are the dental office and the waiting room accessible for patients with special needs?	From 0: Not at all accessible to 10: Very accessible	8.69<5 91.31>5
11.- Which of these treatments do you feel capable of and comfortable performing on a patient with Down Syndrome?	I do not treat patients with DS Oral examination, oral cleaning or giving cleaning instructions Make seals, plugs or remove parts Perform endodontic treatments	7.64 60.6 63.4 26.72

**Table 2:** Statistical analyses of the results between the possible treatments, and the professionals’ age or year of graduation or the professionals’ sex.

VARIABLES	TREATMENT								
	DENTAL PROPHYLAXIS			SEALING-BLACKING-EXTRACTION			ENDODONTIC TREATMENT		
	NO%	YES%	p	NO%	YES%	p	NO%	YES%	p
YEAR OF BIRTH <1960 between 1960-1970 between 1971-1980 between 1981-1990 > 1991	19.20 15.40 28.80 28.80 7.70	5.20 19.50 32.50 36.40 6.50	0.158	14.30 14.30 26.50 32.70 12.20	8.80 20.00 33.70 33.70 3.80	0.279	8.70 17.30 29.80 37.50 6.70	20.00 20.00 36.00 16.00 8.00	0.234
	NO%	YES%	p	NO%	YES%	p	NO%	YES%	p
YEAR OF GRADUATION between 1970-1980 between 1981-1990 between 1991-2000 between 2001-2010 > 2011	3.80 25.00 19.20 36.50 15.40	0.00 11.70 32.50 32.50 23.40	0.04*	0.00 16.30 26.50 36.70 20.40	2.50 17.50 27.50 32.50 20.00	0.716	1.90 13.50 27.90 34.60 22.10	0.00 32.00 24.00 32.00 12.00	0.221
	NO%	YES%	p	NO%	YES%	p	NO%	YES%	p
Sex Man Woman	36.50 63.50	23.40 76.60	0.078*	40.80 59.20	21.30 78.80	0.015*	26.90 73.10	36.00 64.00	0.253

**Table 3:** Statistical analysis of the results between the possible treatments and undergraduate academic training, post-graduate academic training and the professional scenarios of the participants.

VARIABLE	TREATMENT								
	DENTAL PROPHYLAXIS			SEALING-BLACKING-EXTRACTION			ENDODONTIC TREATMENT		
	NO%	YES %	p	NO%	YES %	p	NO%	YES %	p
UNDERGRADUATE ACADEMIC TRAINING Without training With training	21.20 78.80	16.90 83.10	0.349	28.60 71.40	12.50 87.50	0.022*	18.3 81.7	20.00 80.00	0.519
	NO%	YES %	p	NO%	YES %	p	NO%	YES %	p
POSTGRADUATE ACADEMIC TRAINING Without training With training	73.10 26.90	72.70 27.30	0.565	87.80 12.20	63.80 36.30	0.002*	78.80 21.20	48.00 52.00	0.003*
	NO%	YES %	p	NO%	YES %	p	NO%	YES %	p
hospital NO YES	96.20 3.80	92.20 7.80	0.302	89.80 10.20	93.20 3.80	0.137	93.30 6.70	96.00 4.00	0.137
	NO%	YES %	p	NO%	YES %	p	NO%	YES %	p
SEVERAL professionals NO YES	38.50 61.50	31.20 68.80	0.252	30.60 69.40	36.30 63.70	0.323	32.70 67.30	40.00 60.00	0.320
	NO%	YES%	p	NO%	YES %	p	NO%	YES %	p
work alone NO YES	69.20 30.80	70.10 29.90	0.532	73.50 26.50	67.50 32.50	0.304	74.00 26.00	52.00 48.00	0.03*

**Table 4:** Multivariate multinomial logistic regression study.

VARIABLE	TREATMENT		
	DENTAL PROPHYLAXIS	SEALING-BLACKING-EXTRACTION	ENDODONTIC TREATMENT
<1960	1	1	1
between 1960-1970	ns	ns	ns
between 1971-1980	ns	ns	ns
between 1981-1990	ns	ns	ns
> 1991	ns	ns	ns
Man	1	1	1
Woman	ns	ns	ns
between 1970-1980	1	1	1
between 1981-1990	ns	ns	ns
between 1991-2000	ns	ns	ns
between 2001-2010	ns	ns	ns
> 2011	ns	ns	ns
Degree no	1	1	1
Degree yes	ns	ns	ns
Postgraduate not	1	1	1
Postgraduate yes	ns	7.48 (2.19-25.45)	3.26 (1.08-9,79)
Hospital yes	1	1	1
Hospital no	ns	ns	ns
Several do not	1	1	1
Several yes	ns	ns	ns
Only	1	1	1
Not only	ns	ns	ns

## Data Availability

The datasets used and/or analyzed during the current study are available from the corresponding author.
